# Usefulness of daily monitoring of procalcitonin and C-reactive protein in the early diagnosis of infection after elective colonic surgery

**DOI:** 10.1186/cc10635

**Published:** 2012-03-20

**Authors:** J Rebanda, P Povoa

**Affiliations:** 1 Hospital São Francisco Xavier, CHLO, Lisbon, Portugal

## Introduction

The diagnosis of infectious complications after elective colonic surgery is frequently misleading, delaying its resolution. Recently several biomarkers, namely procalcitonin (PCT), have been described as more specific in infection diagnosis.

## Methods

We conducted a prospective observational study segregating patients submitted to elective colonic surgery. Patients were assessed before surgery, and then from the day of surgery until discharge or the 12th day. C-reactive protein (CRP) and PCT were measured daily. We compared infected and noninfected patients.

## Results

A total of 50 patients were included during a 12-month period (age 70.5 ± 9.4 years, 50% male). The 21 patients (42%) that subsequently developed infection (16 surgical wound infections) had age, Charlson comorbidity score, primary diagnosis, surgical procedure, intestinal preparation and antibiotic prophylaxis similar to those who had an uneventful recovery. Infection was less frequent in men (28% vs. 72%, *P *= 0.042). Moreover PCT and CRP before surgery were equally low in patients with or without postoperative infection (0.10 ± 0.06 vs. 0.07 ± 0.04 ng/ml; 1.81 ± 2.83 vs. 0.72 ± 1.12 mg/dl, respectively). After surgery, both PCT and CRP increased markedly: PCT increased around 10× the basal level and peaked at 24 to 48 hours; CRP increased more than 15× and peaked at 48 hours. Infection was diagnosed a median of 7 days after surgery. The CRP time-course from the day of surgery onwards was significantly different in infected and noninfected patients (*P *= 0.001). In opposition, the PCT time-course was almost parallel in both groups (*P *= 0.866). To assess the diagnostic performance of each biomarker, we performed multiple comparisons between infected and noninfected patients between day 5 and day 9. The CRP concentration was significantly different (*P *< 0.01, Bonferroni correction) on days 6, 7 and 8. The area under the ROC curve of CRP of days 6, 7 and 8 were 0.74, 0.73 and 0.75, respectively. A CRP concentration >5.0 mg/dl at day 6 was predictive of infection with a sensitivity of 85% and a specificity of 62% (positive likelihood ratio 2.2, negative likelihood ratio 0.2).

## Conclusion

After a major elective surgical insult both CRP and PCT serum levels increased independently of the presence of infection. The CRP time-course showed to be useful in the early detection of an infectious complication whereas PCT was unhelpful.

